# CircRNA-regulated immune responses of asian honey bee workers to microsporidian infection

**DOI:** 10.3389/fgene.2022.1013239

**Published:** 2022-10-04

**Authors:** Zhiwei Zhu, Jie Wang, Xiaoxue Fan, Qi Long, Huazhi Chen, Yaping Ye, Kaiyao Zhang, Zhongmin Ren, Yang Zhang, Qingsheng Niu, Dafu Chen, Rui Guo

**Affiliations:** ^1^ College of Animal Sciences (College of Bee Science), Fujian Agriculture and Forestry University, Fuzhou, China; ^2^ Apiculture Science Institute of Jilin Province, Jilin, China; ^3^ Apitherapy Research Institute, Fujian Agriculture and Forestry University, Fuzhou, China

**Keywords:** *Apis cerana cerana*, circular RNA, *Nosema ceranae*, non-coding RNA, immune response, host-pathogen interaction

## Abstract

*Nosema ceranae* is a widespread fungal parasite for honey bees, causing bee nosemosis. Based on deep sequencing and bioinformatics, identification of circular RNAs (circRNAs) in *Apis cerana* workers’ midguts and circRNA-regulated immune response of host to *N. ceranae* invasion were conducted in this current work, followed by molecular verification of back-splicing sites and expression trends of circRNAs. Here, 10185 and 7405 circRNAs were identified in the midguts of workers at 7 days (AcT1) and 10 days (AcT2) post inoculation days post-inoculation with *N. ceranae*. PCR amplification result verified the back-splicing sites within three specific circRNAs (novel_circ_005123, novel_circ_007177, and novel_circ_015140) expressed in *N. ceranae*-inoculated midgut. In combination with transcriptome data from corresponding un-inoculated midguts (AcCK1 and AcCK2), 2266 circRNAs were found to be shared by the aforementioned four groups, whereas the numbers of specific ones were 2618, 1917, 5691, and 3723 respectively. Further, 83 52) differentially expressed circRNAs (DEcircRNAs) were identified in AcCK1 vs. AcT1 (AcCK2 vs. AcT2) comparison group. Source genes of DEcircRNAs in workers’ midgut at seven dpi were involved in two cellular immune-related pathways such as endocytosis and ubiquitin mediated proteolysis. Additionally, competing endogenous RNA (ceRNA) network analysis showed that 23 13) DEcircRNAs in AcCK1 vs. AcT1 (AcCK2 vs. AcT2) comparison group could target 18 14) miRNAs and further link to 1111 (1093) mRNAs. These target mRNAs were annotated to six cellular immunity pathways including endocytosis, lysosome, phagosome, ubiquitin mediated proteolysis, metabolism of xenobiotics by cytochrome P450, and insect hormone biosynthesis. Moreover, 284 164) internal ribosome entry site and 54 26) ORFs were identified from DEcircRNAs in AcCK1 vs. AcT1 (AcCK2 vs. AcT2) comparison group; additionally, ORFs in DEcircRNAs in midgut at seven dpi with *N. ceranae* were associated with several cellular immune pathways including endocytosis and ubiquitin-mediated proteolysis. Ultimately, RT-qPCR results showed that the expression trends of six DEcircRNAs were consistent with those in transcriptome data. These results demonstrated that *N. ceranae* altered the expression pattern of circRNAs in *A. c. cerana* workers’ midguts, and DEcircRNAs were likely to regulate host cellular and humoral immune response to microsporidian infection. Our findings lay a foundation for clarifying the mechanism underlying host immune response to *N. ceranae* infection and provide a new insight into interaction between Asian honey bee and microsporidian.

## Introduction

As a novel player in the world of non-coding RNA (ncRNA), circular RNA (circRNA) has become a worldwide research hotspot. Different from canonical alternative splicing, circRNA is generated by the back-splicing of pre-mRNA (Li et al., 2018). In comparison with linear RNA, circRNA is more resistant to RNase R enzyme digestion owing to its special covalently closed-loop structure; hence, circRNA is regarded as an ideal endogenous biomarker (Meng et al., 2017). CircRNAs are abundant in eukaryotic cells and have versatile functions such as the regulation of source gene transcription (Li et al., 2015), absorption of miRNAs or RNA binding proteins as “molecular sponges” (Han et al., 2020), and translation into peptides or proteins (Wang C. et al., 2020). Accumulating evidence suggests that circRNAs are involved in the occurrence and development of human disease, such as cervical (Chen R. X. et al., 2019) and lung cancers (Wang Z. Y. et al., 2020). The biological function of circRNAs as competing endogenous RNAs (ceRNAs) has only been deeply studied in humans and a few other model species (Han et al., 2020; Li et al., 2020). For example, Li et al. (2020) reported that circTLK1 was highly expressed in renal cell carcinoma (RCC) and could promote RCC progression through the miR-136–5p/CBX4 pathway, and circTLK1 could serve as a diagnostic molecules and therapeutic targets for renal cancer. However, research on insect circRNAs are limited, and the few studies have mainly focused on *Drosophila melanogaster* (Westholm et al., 2014; Huang C. et al., 2018; Krishnamoorthy and Kadener, 2021), *Bombyx mori* (Wang and Wang, 2015; Gan et al., 2017; Hu et al., 2018a; Hu et al., 2018b), and honey bees (Chen X. et al., 2019; Thölken et al., 2019; Chen D. F. et al., 2020). Westholm’s group predicted more than 2 500 circRNAs using transcriptome data from *Drosophila*, and revealed that circRNAs were abundantly expressed in the brain and accumulated over time (Westholm et al., 2014). Wang C. et al. (2020) discovered that *Bombyx mori* CircEgg was mainly located in the cytoplasm and circEgg overexpression inhibited the production of linear transcripts of *BmEgg*, circEgg inhibited methylation of histone H3 lysine nine by acting as a “sponge” of bmo-miR-3391–5p. Our group previously conducted a comprehensive investigation of circRNAs in the midguts of European honey bee *Apis mellifera* and Asian honey bee *Apis cerana*. Chen et al. identified 1101 circRNAs in *Apis mellifera ligustica* workers using a combination of RNA-seq and bioinformatics (Chen D. F. et al., 2020), Xiong et al. analyzed the expression profile of circRNAs during the developmental process of *Apis cerana* workers’ midguts, and uncovered the potential regulatory role of differentially expressed circRNAs (DEcircRNAs) (Xiong et al., 2018).

*Nosema ceranae*, an emergent fungal parasite, infects not only the midgut epithelial cells of adult bees but also bee larvae (Huang and Evans, 2020). It was first identified in *Apis cerana* colonies reared in China (Fries et al., 1996) and then spread to *A. mellifera* colonies in Europe (Higes et al., 2010), America (Emsen et al., 2020), and other parts of the world (Giersch et al., 2009). *N. ceranae* spores enter the midgut of the bee host through ingestion of contaminated food or water and then germinate due to activation by the special physical and chemical conditions inside the midgut. The infective sporoplasm is injected into the host midgut epithelial cell and replicates by stealing host material and energy. As the quantity of spores increases, the host cell finally ruptures, and the released spores in the feces become new sources of infection *via* the feeding and cleaning activities inside the colonies, or are disseminated into the environment (Higes et al., 2007; Gisder et al., 2011). *N. ceranae* infection has a negative influence on bee hosts, such as midgut epithelial cell damage, energy stress, immunosuppression, cell apoptosis inhibition, and lifespan reduction (Antúnez et al., 2009; Mayack and Naug, 2009; Goblirsch et al., 2013; Kurze et al., 2018; Panek et al., 2018). Additionally, *N. ceranae* infection severely weakens health and productivity of bee colonies in conjunction with other biological or environmental stresses (Doublet et al., 2015). *A. c. cerana*, a subspecies of *A. cerana*, is mainly distributed and widely used in Asian countries including China. Compared with *A. mellifera*, *A. cerana* is more adaptive to extreme weather conditions and is good at collecting scattered nectar sources (Zhao et al., 2020). Additionally, *A. c. cerana* has been used as a model for investigating host‒pathogen interactions (Huang S. K. et al., 2018). The reference genome of *A. cerana* (Park et al., 2015) was published in 2015, much later than the *A. mellifera* genome (Honeybee Genome Sequencing Consortium, 2006). Currently, study on the omics and molecular biology of *A. cerana* is lagging when compared with those of *A. mellifera*, and the interaction between *A. cerana* and parasites or pathogens is still largely unknown. Previously, our team investigated the immune response of *A. c. cerana* workers to *N. ceranae* infection (Xing et al., 2021), and deciphered the differential expression profile of host miRNAs during microsporidian infection and DEmiRNA-regulated host response (Chen D. F. et al., 2019).

CircRNAs have been identified in both *A. c. cerana* (Chen D. F. et al., 2020) and *N. ceranae* (Guo et al., 2018b) by our group. CircRNAs have been suggested to be crucial regulators engaged in host-pathogen interactions. However, research on the interaction between Asian honey bees and *N. ceranae* is still lacking. Our group previously conducted deep sequencing of *A. c. ceranae* workers’ midgut tissues at 7 days post-inoculation (dpi) and 10 dpi with *N. ceranae* (AcT1 and AcT2 groups) and corresponding un-inoculated midgut tissues (AcCK1 and AcCK2 groups), and identified 9589 circRNAs using transcriptome data from un-inoculated groups (Chen D. F. et al., 2020). Here, to unclose the circRNA-regulated responses of Asian honey bee workers to *N. ceranae* infection, utilizing the obtained high-quality transcriptome data, the differential expression pattern of circRNAs in *A. c. cerana* workers’ midguts in response to *N. ceranae* invasion was analyzed followed by an in-depth investigation of the host response mediated by DEcircRNAs, with a focus on cellular and humoral immune responses. To the best of our knowledge, this is the first documentation of a circRNA-regulated response of Asian honey bee to microsporidian invasion. The findings in this current work can not only lay a key foundation for clarifying the underlying mechanism but also provide novel insights into Asian honey bee-microsporidian interactions.

## Materials and methods

### Honey bees and microsporidians

Three *A. c. cerana* colonies located in the teaching apiary of the College of Animal Sciences (College of Bee Science) in Fujian Agriculture and Forestry University were used for this study. Microscopic observations and PCR identification verified that these colonies were *N. ceranae*-free. *Varroa* was not observed before and during the experiment. RT-PCR was conducted to detect the prevalence of seven common bee viruses (DWV, KBV, ABPV, CBPV, IAPV, SBV, and BQCV) and two bee microsporidia (*Nosema apis* and *N. ceranae*) in the newly emergent workers based on previously described specific primers (Stoltz et al., 1995; Benjeddou et al., 2001; Ribiere et al., 2002; Genersch, 2005; Chen et al., 2008; Singh et al., 2010; Chen D. F. et al., 2019), and agarose gel electrophoresis (AGE) indicated that no bands specific for the aforementioned viruses and microsporidia were amplified (Chen D. F. et al., 2019; Xing et al., 2021).

Foragers were collected from a *N. ceranae*-infected colony in an apiary in Fuzhou city, Fujian Province, China. *N. ceranae* spores were previously prepared using the Percoll discontinuous centrifugation method by our group (Chen D. F. et al., 2019).

### Source of strand-specific cDNA library-based RNA-seq data

Midgut tissues of *N. ceranae*-inoculated *A. c. cerana* workers at seven dpi and 10 dpi and corresponding un-inoculated workers’ midguts were previously prepared by our team (Xing et al., 2021). There were three biological replicates for each treatment group or control group. RNA isolation, cDNA library construction, deep sequencing, and data quality control were previously conducted (Xing et al., 2021). The 12 constructed cDNA libraries were sequenced on an Illumina HiSeq 4000 platform (Illumina). Raw data are available in the NCBI Short Read Archive database (http://www.ncbi.nlm.nih.gov/sra/) under BioProject number: PRJNA406998. In total, 1,809,736,786 raw reads were produced from RNA-seq and 1,562,162,742 clean reads were obtained after quality control. The mean Q20 values of clean reads from the control groups and treatment groups were 94.76% and 94.77%, respectively, and the mapping ratios of clean reads to the reference genome of *A. cerana* were 75.78% (AcCK1), 55.01% (AcCK2), 78.13% (AcT1) and 44.19% (AcT2) (Chen D. F. et al., 2020; Fu et al., 2020). High-quality strand-specific cDNA library-based RNA-seq data could be utilized for circRNA identification and DEcircRNA investigation in this work.

### sRNA-seq data source

In another previous study, midgut tissues of *N. ceranae*-inoculated *A. c. cerana* workers at seven dpi and 10 dpi and corresponding un-inoculated workers’ midguts were prepared by our team (Chen D. F. et al., 2019). Three biological replicates were performed for each treatment group or control group. RNA extraction, cDNA library construction, sRNA-seq, and data quality control were previously performed (Chen D. F. et al., 2019). The 12 constructed cDNA libraries were subjected to sequencing on an Illumina MiSeq platform with the single-end strategy. A total of 127,523,419 raw reads were generated from the sRNA-seq data, and 122,104,443 clean reads were obtained after quality control. The Pearson correlation between every replica in each group was above 0.9619 (Chen D. F. et al., 2019). Thus, the high-quality sRNA-seq data could be used for target prediction and regulatory network construction of DEcircRNAs in this study.

### Bioinformatic prediction of circRNAs

CircRNAs in the AcCK1 and AcCK2 groups were identified in a previous study (Chen D. F. et al., 2020). In this work, circRNAs in the AcT1 and AcT2 groups were identified following our previously described method (Chen D. F. et al., 2020). Briefly, firstly, clean reads were mapped to the *A. cerana* reference genome (assembly ACSNU-2.0) using TopHat software (Kim et al., 2013); secondly, 20 nt at both ends of unmapped reads were then extracted and independently mapped to the reference genome; thirdly, the mapped anchor reads were submitted to find_circ software (Memczak et al., 2013) to perform circRNA identification according to the following criteria: circRNA length <100 kb, best qual A > 35 or best qual B > 35, anchor overlap ≤2, n uniq >2, edit ≤2, n uniq > int (samples/2), and breakpoints = 1.

### Identification of DEcircRNAs

The expression level of each circRNA was normalized to the mapped back-splicing junction reads per million (RPM) mapped-reads value. Following the threshold |FC (fold change)| ≥ 2, *p* value <0.05, and false discovery rate (FDR) ≤ 1, DEcircRNAs in the AcCK1 vs AcT1 and AcCK2 vs AcT2 comparison groups were identified using DESeq software (Wang et al., 2010).

### Analysis of the source genes of DEcircRNAs

CircRNAs can regulate the expression of source genes *via* interactions with RNA polymerase II, U1 ribonucleoprotein, and gene promoters (Zhang et al., 2013; Li et al., 2015). According to the method described by Chen D. F. et al (2020). The source genes of DEcircRNAs were predicted by mapping the anchor reads at both ends of DEcircRNAs to the *A. cerana* reference genome (assembly ACSNU-2.0) using Bowtie software. Gene Ontology (GO) term analysis of the circRNAs’ source genes was conducted with the DAVID tool (http://david.abcc.ncifcrf.gov/) (Huang et al., 2007), and the GO categories were clarified using a two-sided Fisher’s exact test, while the FDR was calculated to correct the *p* value (Jung, 2014). A Kyoto Encyclopedia of Genes and Genomes (KEGG) pathway analysis was performed according to the annotation of the KEGG database (http://www.genome.jp/kegg/) (Du et al., 2014).

### Construction and investigation of DEcircRNA-miRNA and DEcircRNA-miRNA‒mRNA regulatory networks

In combination with previously identified miRNAs based on sRNA-seq data (Chen D. F. et al., 2019), target miRNAs of DEcircRNAs were predicted following a previously described protocol by Chen D. F. et al. (2020). Briefly, according to the criteria of *p* ≤ 0.05 and free energy ≤ −35 kcal/mol, potential target miRNAs of DEcircRNAs were predicted using three software programs, namely, mireap, miranda (v3.3a) and TargetScan (version: 7.0), followed by construction of the DEcircRNA-miRNA regulatory network; subsequently, target mRNAs of DEcircRNA-targeted miRNAs were further predicted, and then the DEcircRNA-miRNA-mRNA regulatory network was constructed; finally, the regulatory networks were visualized by Cytoscape software (Smoot et al., 2011) with the default parameters. GO term and KEGG pathway investigation of the target mRNAs was further conducted using the abovementioned methods.

### RT‒PCR and sanger sequencing of circRNAs

Three circRNAs (novel_circ_005123, novel_circ_007177, and novel_circ_015140) shared by the AcT1 and AcT2 groups were randomly selected for molecular verification. Following our previously described method (Chen D. F. et al., 2020), divergent primers for these circRNAs (Table 1) were designed using DNAMAN eight software (Lynnon Biosoft, United States) and then synthesized by Shanghai Sangon Biological Co., Ltd. Total RNA of *N. ceranae*-inoculated workers’ midguts at seven dpi and 10 dpi was extracted with an AxyPre RNA extraction kit (Axygen, China), and then digested with 3 U/mg RNase R (Geneseed, China) at 37°C for 15 min to remove linear RNA. Next, the first-strand cDNAs were synthesized *via* reverse transcription with random primers. The PCR system was 20 μl in volume and contained 1 μl of template, 10 μl of Mixture (Yeasen, China), 1 μl upstream primers (10 μmol/L), 1 μl downstream primers (10 μmol/L), and 7 μl ddH_2_O. The reaction was carried out on a T100 thermocycler (Bio-Rad, United States) under the following conditions: 94°C for 5 min; followed by 36 cycles of 94°C for 40 s, an appropriate annealing temperature (according to the melting temperature of the primers) for 30 s; 72°C for 30 s; and 72°C for 5 min. The PCR products were detected on 1.5% agarose gel electrophoresis (AGE) followed by TA cloning and Sanger sequencing.

**TABLE 1:** Primers for RT-PCR and RT-qPCR conducted in this work.

Forward primer	Sequence (5′-3′)	Reverse primer	Sequence (5′-3′)	Purpose
novel_circ_005123-F	AGT​GGA​GGA​TTG​CTG​GGT​AG	novel_circ_005123-R	GCT​TTG​ACA​GTC​GTA​TTC​GG	RT-PCR
novel_circ_007177-F	GCA​AGC​AAA​GCA​TCG​TTA​C	novel_circ_007177-R	AAT​ACT​GCC​AGG​TTC​TCA​CAG	RT-PCR
novel_circ_015140-F	CCT​TCA​ATG​TCT​CCC​TCT​GTC	novel_circ_015140-R	TGG​CAC​TAC​GAC​CAA​ATC​C	RT-PCR
novel_circ_016536-F	ATC​TCC​TAC​TTC​GCA​CTG​GG	novel_circ_016536-R	ATC​GTA​TCA​CTT​CCC​TCG​C	RT-PCR
novel_circ_010689-F	GCT​CTC​GTT​TAC​CTC​TTC​AGA	novel_circ_010689-R	CGC​TAT​CTT​CTC​CAC​TAT​TTG​G	RT-qPCR
novel_circ_005734-F	GGA​GGC​TAT​CCG​AGA​TGA​T	novel_circ_005734-R	CTT​CGT​TGG​TGG​TGA​CTT​C	RT-qPCR
novel_circ_016924-F	TCG​GGA​CGG​TAG​CAG​TAA​T	novel_circ_016924-R	CAG​TGG​TAT​CCT​CGT​GTC​GT	RT-qPCR
novel_circ_017693-F	CAC​TGT​CGT​GGT​AGC​CAA​A	novel_circ_017693-R	GGG​AAG​AAC​CTG​GAA​CAT​C	RT-qPCR
novel_circ_008642-F	TAC​GGG​ACA​GCG​AGA​AGT​T	novel_circ_008642-R	CGT​GTA​TCC​AAT​CAT​CAC​CG	RT-qPCR
novel_circ_005927-F	AGT​TGC​CGT​AAA​TGG​TGT​C	novel_circ_005927-R	CGA​CTC​GGT​TCT​TCC​AAA​T	RT-qPCR
*actin-F*	GGT​TGT​TGA​TAG​TGG​AGA​TGG	*actin*-R	CAC​GAC​CAG​CAA​TAG​GAA​T	RT-qPCR

### RT-qPCR validation of DEcircRNAs

Four DEcircRNAs in AcCK1 vs AcT1 comparison group (novel_circ_010689, novel_circ_005734, novel_circ_016924, novel_circ_0176939) and two DEcircRNAs in AcCK2 vs. AcT2 comparison group (novel_circ_008642, novel_circ_005927) were randomly selected for RT-qPCR. Divergent primers for these DEcircRNAs were designed and synthesized **(** Table 1 **)**. Total RNA from the AcCK1, AcT1, AcCK2 and AcT2 groups was isolated and then subjected to reverse transcription. The resulting cDNA was used as a template for the internal control (*actin*) and DEcircRNAs. The RT-qPCR system was 20 μl in a volume containing 1 μl upstream primers (10.0 μmol/L), 1 μl downstream primers (10.0 μmol/L), 1 μl cDNA, 10 μl SYBR Green Dye, and 7 μl DEPC H_2_O. RT-qPCR was performed on an ABI Q3 Real-time PCR Detection System (Applied Biosystems, United States) under the following conditions: predenaturation step at 94°C for 5 min; 36 amplification cycles of denaturation at 94°C for 50 s, annealing at 60°C for 30 s, and elongation at 72°C for 1 min; and a final elongation step at 72°C for 30 s. The data were calculated using the 2^-△△Ct^ method and presented as relative expression levels from three parallel replicates and three biological replicates, followed by analysis and visualization using GraphPad Prism 6.0 software (GraphPad, United States).

### Statistical analysis

Statistical analyses were conducted utilizing SPSS 16.0 (IBM, Armonk, NY, United States) and GraphPad Prism 6.0 (GraphPad, United States) software. Data were shown as the mean ± standard deviation (SD). Statistical analyses were performed on basis of an independent-sample *t* test. Fisher’s exact test was conducted to filter the significant GO terms and KEGG pathways with R software 3.3.1 (R Development Core Team, https://www.r-project.org/). *p* < 0.05 was considered statistically significant.

## Results

### Identification, investigation, and validation of circRNAs in *N. ceranae*-inoculated *A. C. ceranae* workers’ midguts

Totally, 524100096 and 615893838 raw reads were generated from the AcT1 and AcT2 groups, and 515604182 and 601712328 clean reads were obtained after quality control, respectively; additionally, 338500740 and 570789154 anchor reads were identified, among which 31889778 (28.28%) and 35037206 (20.60%) were respectively aligned to the reference genome of *A. cerana*.

**FIGURE 1:**
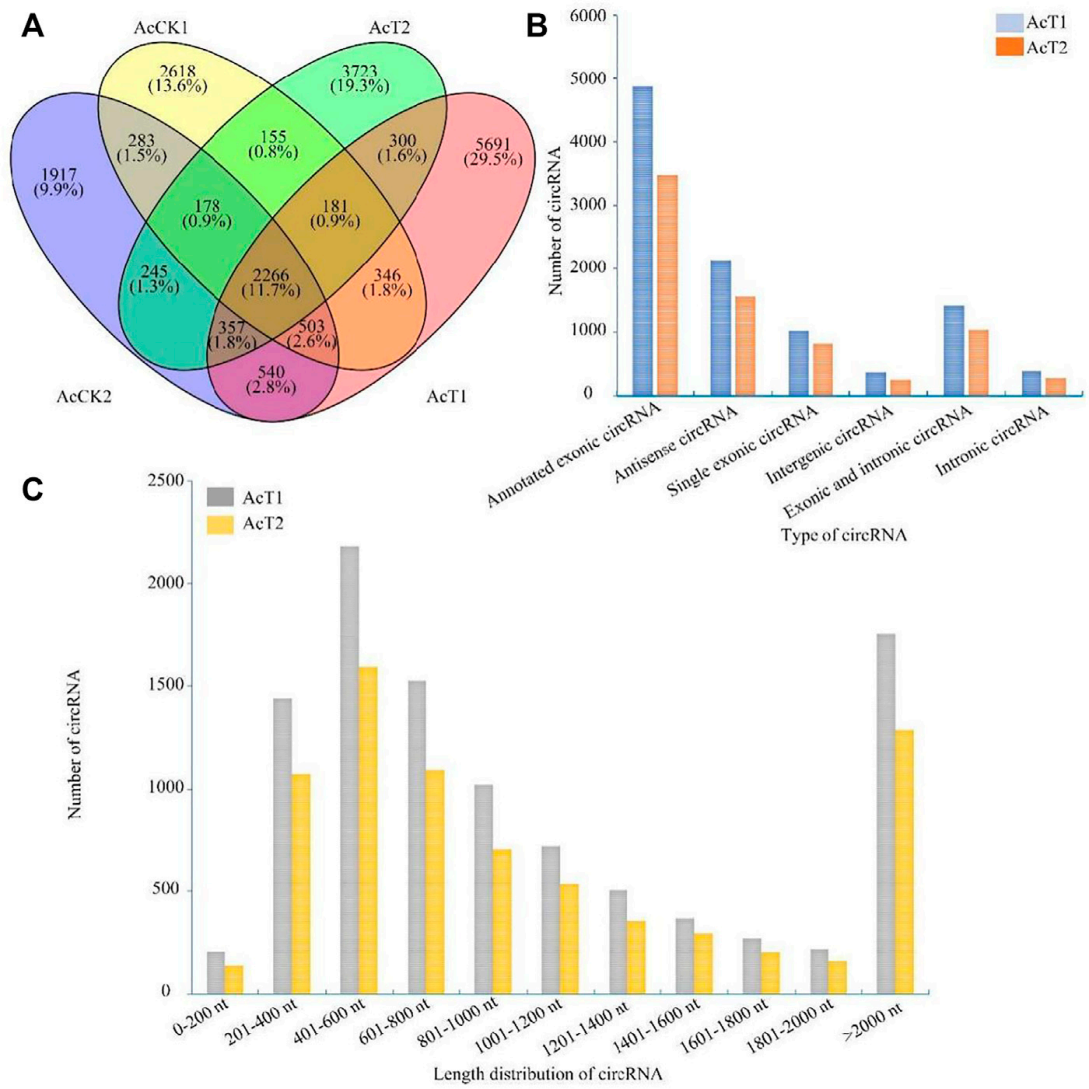
Number, type, and length distribution of circRNAs in the midguts of *A. c. cerana* workers inoculated with *N. ceranae*. **(A)** Venn diagram of circRNAs in AcCK1, AcCK2, AcT1, and AcT2 groups. **(B)** Number statistics of circRNAs derived from various genomic origins. **(C)** Length distribution of circRNAs.

In the AcT1 and AcT2 groups, 10185 and 7405 circRNAs were identified respectively. Combined with the previously identified circRNAs in the un-inoculated groups, the Venn analysis indicated that 2266 circRNAs were shared by the AcCK1, AcCK2, AcT1, and AcT2 groups, while 2618, 1917, 5691 and 3723 ones were specific to each group, respectively (Figure 1A). Additionally, annotated exonic circRNA was the most abundant type in both the AcT1 and AcT2 groups, followed by antisense circRNA and single exonic circRNA (Figure 1b). Moreover, the length of circRNAs in the *N. ceranae*-inoculated groups ranged from 1 nt to more than 2000 nt, and circRNAs with a length distribution of 401–600 nt were the most abundant (Figure 1C).

**FIGURE 2:**
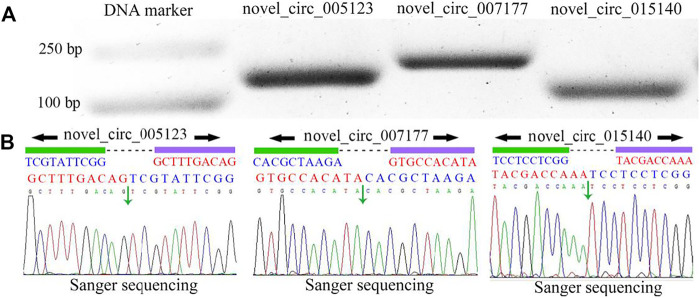
PCR amplification **(A)** and Sanger sequencing **(B)** of three *A. c. cerana* circRNAs. Black arrows indicate the direction of primers for PCR amplification, and green arrows indicate back-splicing sites within circRNAs.

PCR amplification was performed to further validate the three specific circRNAs identified in *N. ceranae*-inoculated midguts, and AGE suggested that fragments with expected sizes could be amplified using specific divergent primers for novel_circ_005123 (approximately 162 bp), novel_circ_007177 (approximately 195 bp), and novel_circ_015140 (approximately 108 bp) (Figure 2A). Additionally, the back-splicing sites of these selected circRNAs were successfully detected using Sanger sequencing (Figure 2B).

### Differential expression pattern of circRNAs involved in the host response to *N. ceranae* infection

**FIGURE 3:**
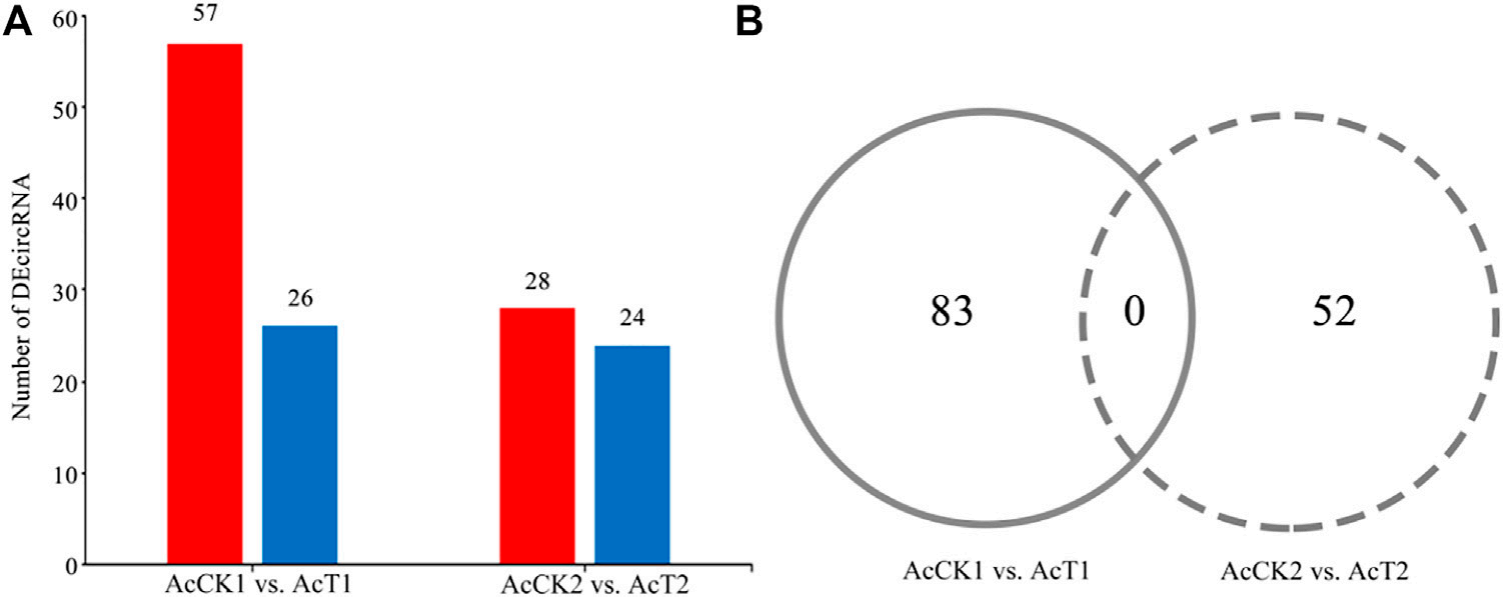
Number statistics of DEcircRNAs in AcCK1 vs. AcT1 and AcCK2 vs. AcT2 comparison groups. **(A)** Number of up- and down-regulated circRNAs **(B)** Venn analysis of DEcircRNAs.

A total of 83 DEcircRNAs were identified in the AcCK1 vs AcT1 comparison group, including 57 up-regulated circRNAs and 26 down-regulated circRNAs **(** Figure 3A, see also Supplementary Table S1. The expression levels of the DEcircRNAs were between 0.001 and 353.49, and the most up-regulated and down-regulated circRNAs were novel_circ_012754 (log_2_FC = 18.16) and novel_circ_017486 (log_2_FC = -17.05), respectively. Comparatively, 28 up-regulated circRNAs and 24 down-regulated circRNAs were detected in the AcCK2 vs. AcT2 comparison group **(** Figure 3A **,** see also Supplementary Table S2. The expression levels of the circRNAs were among 0.001–445.77, and the most up-regulated and down-regulated circRNAs were novel_circ_002265 (log_2_FC = 18.77) and novel_circ_011100 (log_2_FC = −17.50), respectively. In addition, none of the DEcircRNAs were shared by the two abovementioned comparison groups (Figure 3B).

### GO term and KEGG pathway analyses of the source genes of the host DEcircRNAs

**FIGURE 4:**
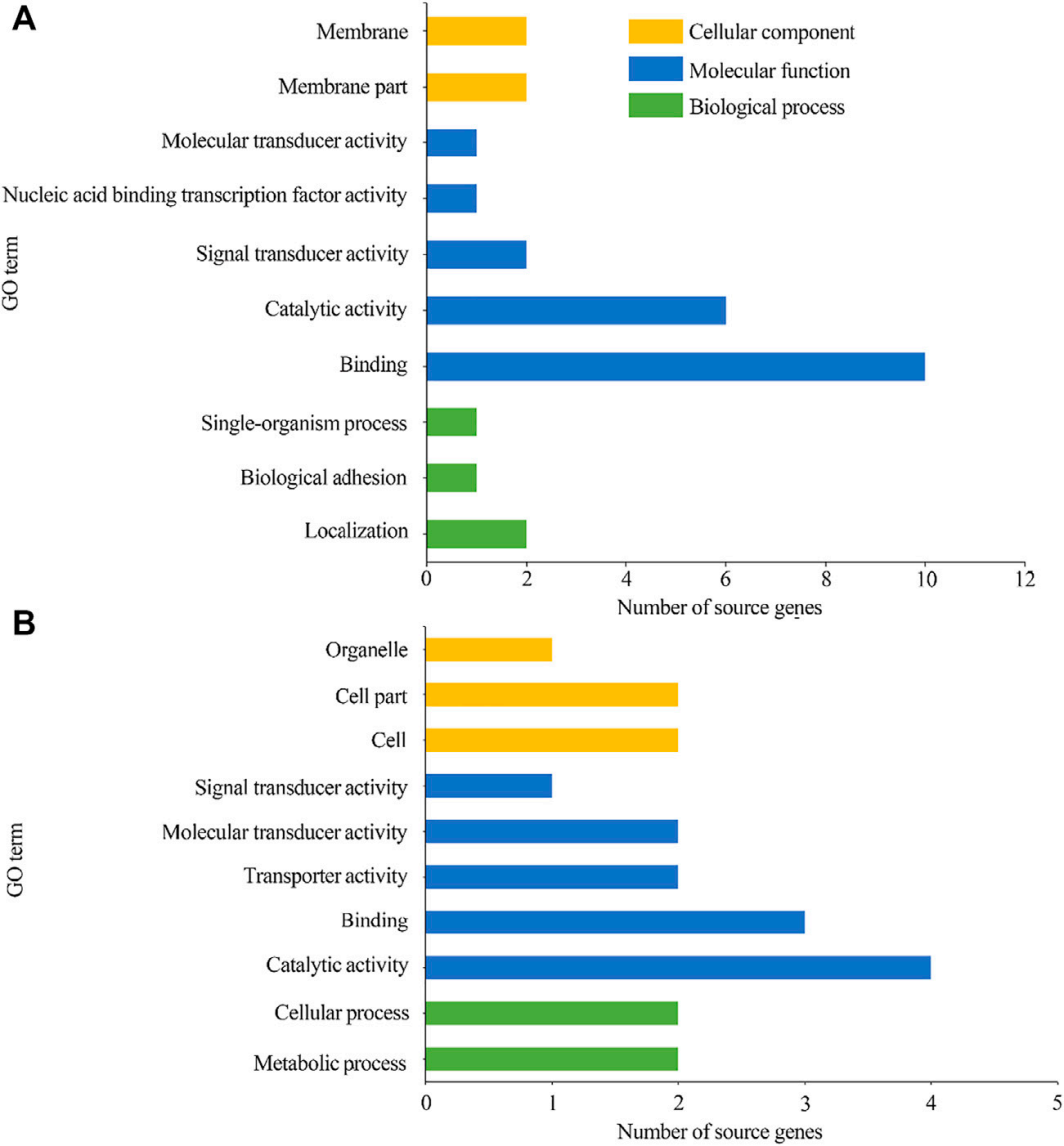
GO categorization of DEcircRNAs’ source genes in AcCK1 vs AcT1 **(A)** and AcCK2 vs AcT2 **(B)** comparison groups.

GO classification suggested that 78 source genes of DEcircRNAs in the AcCK1 vs AcT1 comparison group were predicted; among these, 23 ones were classified into 10 functional terms associated with molecular functions, cellular components, and biological processes, such as binding, localization, and membrane (Figure 4A). Additionally, 45 source genes of DEcircRNAs in the AcCK2 vs. AcT2 comparison group were predicted, among which 13 ones were grouped into 10 functional terms including catalytic activity, cell part, and metabolic processes (Figure 4B).

The KEGG pathway analysis suggested that the source genes of DEcircRNAs in the AcCK1 vs AcT1 comparison group were involved in 12 pathways, such as endocytosis and the Hippo signaling pathway (Table 2). Comparatively, the source genes of DEcircRNAs in the AcCK2 vs. AcT2 comparison group were engaged in five pathways, including the sphingolipid metabolism and the mTOR signaling pathways (Table 3).

**TABLE 2:** KEGG pathways enriched by source genes of DEcircRNAs in AcCK1 vs AcT1 comparison group.

Pathway	Ko number	Number of source gene	Gene id
Spliceosome	ko03040	2	107997744, 108004399
Endocytosis	ko04144	2	107993574, 108001896
Mucin type O-glycan biosynthesis	ko00512	1	108003572
Tyrosine metabolism	ko00350	1	107994400
Other glycan degradation	ko00511	1	108001667
ECM-receptor interaction	ko04512	1	107997219
Insulin resistance	ko04931	1	107999877
FoxO signaling pathway	ko04068	1	107999607
Hippo signaling pathway-fly	ko04391	1	107995510
Ribosome biogenesis in eukaryotes	ko03008	1	107995090
Ubiquitin mediated proteolysis	ko04120	1	108001789
RNA transport	ko03013	1	107998561

**TABLE 3:** KEGG pathways enriched by source genes of DEcircRNAs in AcCK2 vs. AcT2 comparison group.

Pathway	Ko number	Number of source gene	Source gene id
Sphingolipid metabolism	ko00600	1	107995902
ECM-receptor interaction	ko04512	1	107998342
mTOR signaling pathway	ko04150	1	107997811
Insulin resistance	ko04931	1	107999331
Neuroactive ligand-receptor interaction	ko04080	1	107993584

### DEcircRNA-miRNA regulatory network associated with the *N. ceranae* response of *A. C. cerana* workers

**FIGURE 5:**
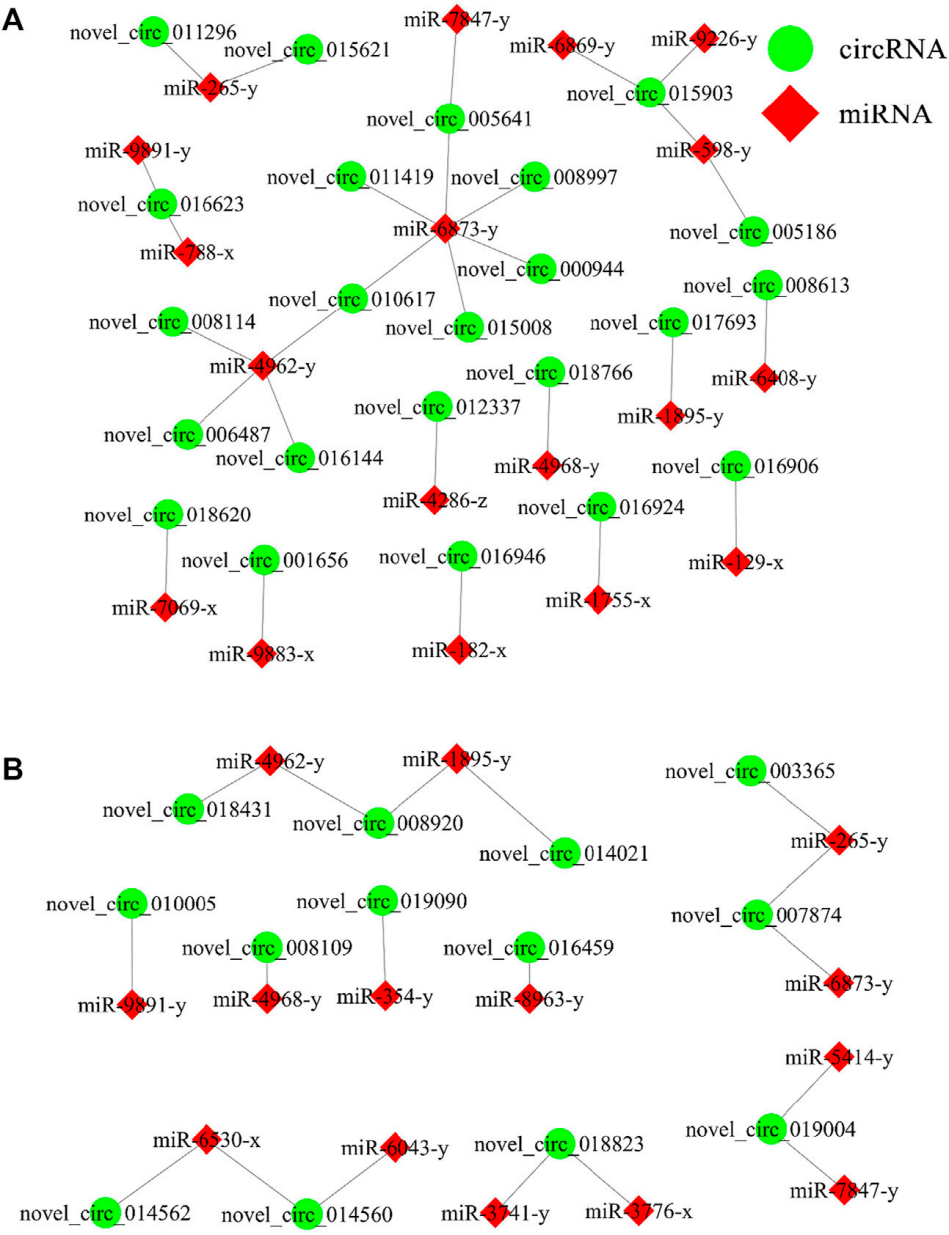
DEcircRNA-miRNA network engaged in *N. ceranae*-response of *A. c. cerana* workers. **(A)** Regulatory network in AcCK1 vs AcT1 comparison group **(B)** Regulatory network in AcCK2 vs AcT2 comparison group.

In the AcCK1 vs. AcT1 comparison group, 23 DEcircRNAs were predicted to target 18 miRNAs; among these DEcircRNAs, novel_circ_015903 could target three miRNAs, novel_circ_016623 and novel_circ_010617 could target two miRNAs, whereas another 20 DEcircRNAs could target only one miRNA (Figure 5A). In addition, 13 DEcircRNAs in the AcCK2 vs. AcT2 comparison group were found to target 14 miRNAs; among these DEcircRNAs, five DEcircRNAs could target two miRNAs, while another eight DEcircRNAs could only target one miRNA (Figure 5B).

### DEcircRNA-miRNA-mRNA regulatory network engaged in the host response to *N. ceranae* infestation

Further investigation showed that 23 DEcircRNAs in the AcCK1 vs AcT1 comparison group can target 18 miRNAs and further target 1111 mRNAs Supplementary Table S3. These target mRNAs were annotated to 10 cellular component-related terms, such as cell and membrane; eight molecular function-related terms, such as catalytic activity and transporter activity; and 11 biological process-related terms, such as cellular process and biological regulation Supplementary Table S5. Additionally, these target mRNAs could also be annotated to 72 pathways, including endocytosis, RNA transport, and ubiquitin-mediated proteolysis Supplementary Table S6. Comparatively, 13 DEcircRNAs in the AcCK2 vs AcT2 comparison groups can target 14 miRNAs and further target 1093 mRNAs Supplementary Table S4. These target mRNAs were involved in 10 cellular component-associated terms, including organelle and cell; eight molecular function-associated terms, including binding and catalytic activity; and 11 biological process-associated terms, including biological regulation and single-organism process Supplementary Table S7. In addition, these target mRNAs could also be annotated to 72 pathways, such as endocytosis, purine metabolism, RNA transport, protein processing of the endoplasmic reticulum, and ubiquitin-mediated proteolysis Supplementary Table S8. Moreover, target mRNAs in both the AcCK1 vs AcT1 and AcCK2 vs AcT2 comparison groups were relevant to six cellular immune-related pathways, including endocytosis, lysosomes, phagosomes, ubiquitin-mediated proteolysis, metabolism of xenobiotics by cytochrome P450, and insect hormone biosynthesis (Table 4); however, no target was found to annotate to any humoral immune pathway.

**TABLE 4:** Summary of cellular immune pathways enriched by DEcircRNA-targeted mRNAs within ceRNA networks.

Pathway	Number of target mRNA in AcCK1 vs AcT1	Number of target mRNA in AcCK2 vs AcT2	Ko number
Endocytosis	28	28	ko04144
Lysosome	4	4	ko04142
Phagosome	3	3	ko04145
Ubiquitin mediated proteolysis	2	2	ko04120
Metabolism of xenobiotics by cytochrome P450	1	1	ko00980
Insect hormone biosynthesis	1	2	ko00981

In total, 284 IRESs and 54 ORFs were identified from the DEcircRNAs in the AcCK1 vs. AcT1 comparison group. Supplementary Table S9, S10. These ORFs were involved in two biological process-associated terms, four molecular function-associated terms, and two cellular component-associated terms Additionally, these ORFs were associated with eight pathways, namely, endocytosis, other glycan degradation, ECM-receptor interaction, insulin resistance, other glycan degradation, FoxO signaling pathway, ribosome biogenesis in eukaryotes, ubiquitin-mediated proteolysis, and spliceosome Supplementary Table S11. Comparatively, 164 IRES and 26 ORF were identified from the DEcircRNAs in the AcCK2 vs AcT2 comparison group Supplementary Table S12. These ORFs were enriched in two biological process-related terms, four molecular function-related terms, and two cellular component-related terms Supplementary Table S13. In addition, these ORFs were relative to four pathways involving sphingolipid metabolism, ECM-receptor interaction, insulin resistance, and neuroactive ligand‒receptor interaction Supplementary Table S14.

### RT-qPCR validation of DEcircRNAs

**FIGURE 6:**
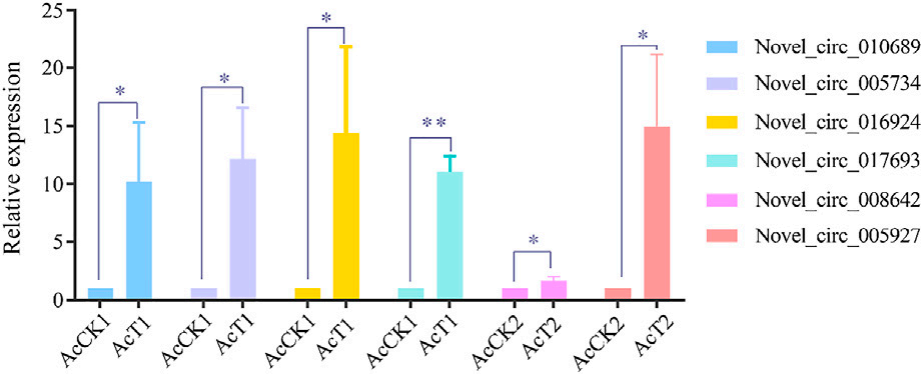
RT-qPCR verification of DEcircRNAs.Student’s t test, “*” indicates *p* ≤ 0.05 and “**” indicates *p* ≤ 0.01.

Six DEcircRNAs were randomly selected for RT-qPCR validation, and the results showed that their expression trends were consistent with those in high-throughput sequencing data, which confirmed the reliability of the transcriptome data used in this current work (Figure 6).

**FIGURE 7:**
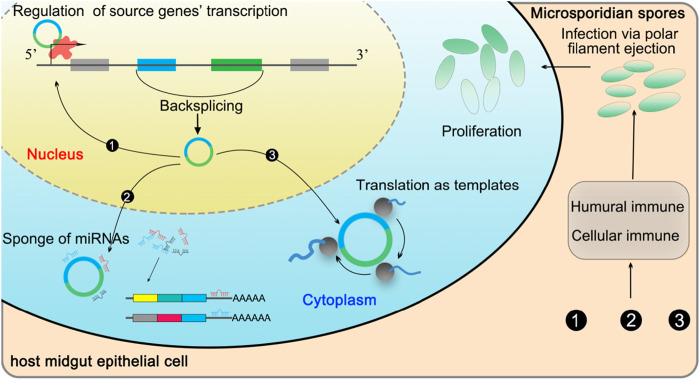
A working model of DEcircRNA-regulated response of *A. c. cerana* workers to *N. ceranae* infection.

Combining findings in this current work, a working model of DEcircRNA-regulated response of *A. c. cerana* workers to *N. ceranae* infection was summarized and presented in Figure 7.

## Discussion

### CircRNAs were abundantly expressed in *A. C. cerana* workers’ midguts and their expression pattern altered during *N. ceranae* infection

Here, based on previously obtained high-quality transcriptome data from *N. ceranae*-inoculated and un-inoculated midguts of *A. c. cerana* workers, the regulatory roles of circRNAs involved in the *N. ceranae*-response of *A. c. cerana* workers were investigated for the first time. In *N. ceranae*-inoculated workers’ midguts at seven dpi and 10 dpi, 10185 and 7405 circRNAs were respectively identified, among which annotated exon circRNA was the most abundant type (Figure 1b); additionally, the length distribution of the identified *A. c. cerana* circRNAs were ranged from 201 nt to 800 nt (Figure 1C). Similarly, we previously identified 6530 and 6289 circRNAs in un-inoculated workers’ midguts using the same bioinformatic approach, and found that their length distribution was among 201 nt∼800 nt and the most abundant type was also annotated exon circRNA (Chen H. Z. et al., 2020). Further analysis showed that the number of circRNAs enriched in each length or circularization type in the *N. ceranae*-inoculated groups was higher than that in the un-inoculated groups, implying that *A. c. cerana* workers may respond to *N. ceranae* infection by altering circRNAs’ length and circularization type. In addition, 2266 circRNAs were shared by the AcCK1, AcCK2, AcT1, and AcT2 groups, while the numbers of specific circRNAs were 2618, 1917, 5717, and 3742, respectively (Figure 1A), indicative of the developmental stage- and stress stage-specific expression of *A. c. cerana* circRNAs, similar to the expression characteristic of circRNAs identified in other animals and plants (Hu et al., 2018a; Liang et al., 2019). Since the information about circRNAs in Asian honey bees is scarse, the circRNAs discovered here could well enrich the reservoir of *A. cerana* circRNAs. Furthermore, 83 and 52 DEcircRNAs were identified circRNAs in the AcCK1 vs. AcT1 and AcCK2 vs AcT2 comparison groups, respectively (Figure 3B), which suggested that the expression profile of circRNAs in host midguts was altered due to *N. ceranae* infection. It is inferred that these host-derived DEcircRNAs were involved in the *N. ceranae*-response. Interestingly, it is noticed that no DEcircRNA was shared by the aforementioned two comparison groups, suggesting that different circRNAs may be utilized by host to respond to the same microsporidian at different timepoints during infection, which deserved more efforts and further investigation. *Apis mellifera ligustica*, a subspecies of western honey bee (*Apis mellifera*), is widely reared bee species in China and many other countries. Recently, we discovered that nine up-regulated and 10 down-regulated circRNAs were shared by *Apis mellifera ligustica* workers’ midguts at seven dpi and 10 dpi with *N. ceranae* (Chen et al., 2022). Together, the results indicated that some circRNAs were adopted by western honey bee at various timepoints during the *N. ceranae* infection, reflecting the difference of circRNA-regulated strategies used by Asian honey bee and western honey bee.

### DEcircRNAs putatively modulated cellular renewal and immune response of *A. C. cerana* workers to *N. ceranae* infection by regulating transcription of source genes

Accumulating evidence suggests that circRNAs are able to exert their functions by regulating the transcription of source genes (Zhang et al., 2013; Li et al., 2015). To steal host cell-produced material and energy for fungal proliferation, *N. ceranae* prolongs the survival time of infected cells by inhibiting apoptosis of *A. mellifera* midgut epithelial cells (Kurze et al., 2018; Paris et al., 2018). Additionally, *N. ceranae* could also cause structural damage to midgut epithelial cells of *A. mellifera* (Panek et al., 2018). The Hippo signaling pathway plays a crucial role in regulating cell proliferation as well as organ growth and regeneration (Tang et al., 2022). Emerging evidence demonstrated the participation of the Hippo signaling pathway in the regulation of immune defense in mammals and insects (Buchon et al., 2009; Hong et al., 2018). In this work, it is detetcted that in the AcCK1 vs AcT1 comparison group 78 source genes of 83 DEcircRNAs (novel_circ_008114) were involved in the Hippo signaling pathway, suggesting the roles of these DEcircRNAs in detecting damage to the midgut epithelial cell structure caused by *N. ceranae* infection. It is speculated that the corresponding DEcircRNAs were employed by the host to regulate source gene transcription, thereby further modulating the Hippo signaling pathway to facilitate cell renewal and regulate the immune response.

Insects defend against pathogenic microorganisms based on cellular and humoral immunity, with the former involving endocytosis, phagocytosis, enzymatic hydrolysis, melanization, and antimicrobial peptide synthesis and release (Lavine and Strand. 2002). In honey bees, endocytosis is a main cellular immune pathway (Aronstein and Murray. 2010). Clathrin-mediated endocytosis is one of the most clearly studied endocytosis pathways. Kim et al. (2009) discovered that when compared with wild-type cells, deletion of *FgEnd1* gene in *Fusarium graminearum* resulted in a significant downregulation of the endocytic marker FM4-64 and a decrease in the mycelium growth rate . Hodgson et al. (2019) disrupted two early and late endosome marker genes Rab5 and Rab7 in *Drosophila* DL1 cells using RNAi, the results showed that progeny virions of *Autographa californica* multiple nucleopolyhedrovirus (AcMNPV) were significantly reduced. Ubiquitin-mediated proteolysis is a classic cellular immune pathway, that involves the E1 (ubiquitin activase), E2 (ubiquitin binding enzyme) and E3 enzymes (ubiquitin ligase); E3 can specifically recognize different substrates and then bind to the E2 enzyme, which is finally recognized and degraded by the protein enzyme body (Sutovsky, 2003). Here, it is observed that two source genes of novel_circ_005307 and novel_circ_017023 in the AcCK1 vs AcT1 comparison group were engaged in endocytosis, while one source gene of novel_circ_016946 was involved in ubiquitin-mediated proteolysis. Together, these findings suggested that the corresponding DEcircRNAs were likely to regulate the two aforementioned cellular immune pathways by regulating source gene transcription, and then further participate in the response of host to *N. ceranae* infection. Intriguingly, in our previous work, we observed that the source genes of DEcircRNAs in *A m ligustica* workers’ midguts during *N. ceranae* infection were enriched in four cellular immune-related pathways, including endocytosis, ubiquitin-mediated proteolysis, lysosomes, and phagosomes (Chen et al., 2022). Collectively, these results indicated that both *A. c. cerana* and *A m ligustica* workers likely regulate endocytosis and ubiquitin-mediated proteolysis via the control of source gene transcription by DEcircRNAs, further responding to *N. ceranae* invasion, but only *A m ligustica* workers were capable of modulating another two cellular immune pathways lysosomes and phagosomes utilizing differential expression of specific circRNAs. The identified novel_circ_005307, novel_circ_017023, and novel_circ_016946 may be candidate targets for further functional study and bee nosemosis control, additional work is required to develop novel circRNA-based control strategies.

### DEcircRNAs potentially regulated cellular immune as well as cell proliferation and apoptosis of *A. C. cerana* workers *via* ceRNA networks during the *N. ceranae* infection

Increasing evidence suggests that circRNAs can regulate target gene expression *via* the ceRNA network and further affect various biological processes, such as the immune response and development (Han et al., 2020; Li et al., 2020). Here, 23 and 13 DEcircRNAs in the AcCK1 vs. AcT1 and AcCK2 vs AcT2 comparison groups were predicted to target 18 and 14 miRNAs and further target 1111 and 1093 mRNAs, respectively, implying that these DEcircRNAs may function as ceRNAs during the host response to *N. ceranae* infection. Further analysis indicated that target mRNAs in the worker’s midgut at seven dpi were associated with six cellular immune pathways including lysosome and phagosome, whereas targets in the worker’s midgut at 10 dpi were involved in five cellular immune-related pathways, namely, endocytosis, ubiquitin-mediated proteolysis, and insect hormone biosynthesis. Interestingly, none of the targets were enriched in the humoral immune pathways. The results demonstrated that the corresponding DEcircRNAs likely regulated the host cellular immune responses to *N. ceranae* infection through ceRNA networks.

The miR-182 gene was abundantly expressed in sensory organs and regulated the development of the retina and inner ear (Wei et al., 2015). Perilli et al. (2019) reported that overexpression of miR-182 in humans could inhibit apoptosis and promote cell proliferation as well as colorectal cancer cell infection by altering tumor cell cycle dynamics and morphology. Sun et al. (2010) revealed that miR-182 regulated RGS17 through two conserved sites within the 3′UTR, and ectopic expression of miR-182 conspicuously inhibited lung cancer cell proliferation and anchorage-independent cell growth. *FOX O 3a* was previously identified as a direct target of miR-182–5p, and miR-182–5p played an inhibitory role in *FOX O 3a* expression. Moreover, activation of the AKT/FOXO3a pathway promoted HCC proliferation and invasive ability, which further resulted in higher death rates (Cao et al., 2018). Wu et al. (2021) observed that miR-182–5p directly targeted the 3′ UTR of the tumor suppresser gene *STARD13*, which significantly relieved the inhibitory effect of decreased miR-182–5p on cell proliferation, migration, and invasion in lung adenocarcinoma. Here, miR-182 was detected to be targeted by novel_circ_016924 (log_2_FC = 17.25, *P*= 0.0020) and novel_circ_016946 (log_2_FC = 16.37, *p* = 0.0077) in the AcCK1 vs. AcT1 comparison group, , indicating that these two DEcircRNAs may play a pivotal role in cell apoptosis and cell proliferation in the *N. ceranae*-response of host by absorbing miR-182. Therefore, miR-182 and its targeted DEcircRNAs were promising biomarkers and molecular targets for diagnosis and control of bee nosemosis frequently occurred in beekeeping industry. In the near future, we plan to conduct overexpression and knockdown of miR-182 and siRNA-based RNAi of corresponding DEcircRNAs following our established technical platforms (Ye et al., 2022; Zhu et al., 2022).

### DEcircRNAs probably regulated endocytosis and ubiquitin-mediated proteolysis in *A. C. cerana* workers’ midguts *via* protein translation during the *N. ceranae* infection

Eukaryotic translation depends on the ribosomal scanning mechanism of the m7G cap structure (Haimov et al., 2015). Due to the lack of a 5’ terminal and poly-A tail, circRNA was previously considered unable to translate proteins. With the rapid development of next-generation and third-generation sequencing technologies, some circRNAs were verified to translate into proteins or small peptides with biological functions using an IRES-based manner (Wang and Wang, 2015; Pamudurti et al., 2017). Yang et al. (2018) reported that FBXW7-185aa, a protein encoded by circRNA FBXW7 (circ-FBXW7), plays an essential part in glioma carcinogenesis and patient clinical prognosis. After transfecting *Drosophila* S2 cells with artificially constructed circRNAs, including the *gfp* gene containing IRES (Wang et al., 2015), Wang and Wang (2015) detected that the constructed circRNA successfully expressed GFP protein. Recently, Weigelt et al. (2020) documented that overexpression of circSfl, a protein-coding circRNA, extended the lifespan of the insulin mutant *Drosophila*. In this current work, novel_circ_017023 and novel_circ_005307 in the AcCK1 vs. AcT1 comparison group were predicted to translate endocytic pathway-related proteins, and novel_circ_016946 was predicted to translate proteins associated with the ubiquitin-mediated proteolysis. The results suggested that the abovementioned three DEcircRNAs were likely to engaged in the cellular immune responses of *A. c. cerana* workers to *N. ceranae* invasion through the protein translation, which deserved further investigation in the future.

## Conclusion

In the present study, we investigated for the first time the expression profiles and potential functions of circRNA in *A. c. cerana* workers’ midguts in response to *N. ceranae* infection. It is demonstrated that the expression pattern of circRNAs was altered due to *N. ceranae* infection and DEcircRNAs may play regulatory roles in the host cellular immune responses through versatile manners, such as regulation of the transcription of source genes,absorption of target miRNAs *via* the ceRNA networks, and translation of proteins as templates. Our data offer a foundation for clarifying the mechanism underlying the immune responses of *A. c. cerana* workers to *N. ceranae* invasion and provide novel insights into host-parasite interactions during bee nosemosis (Figure 7).

## Data Availability

The datasets presented in this study can be found in online repositories. The names of the repository/repositories and accession number(s) can be found in the article/Supplementary Material.
